# Operationalizing the European sustainability competence framework: Development and validation of learning outcomes for GreenComp

**DOI:** 10.12688/openreseurope.20927.1

**Published:** 2025-07-29

**Authors:** Pablo Martín-Ramos, Eva Sánchez-Hernández, Fatma Fourati-Jamoussi, Alice Annelin, Chrysanthi Charatsari, João Ferreira-Santos, Priscila Doran, David Naves-Sousa, Marcia Eugenio-Gozalbo, Lucio Alessandro Lo Giudice, Frederico Oliveira-Pinto, Luis Manuel Navas-Gracia

**Affiliations:** 1 GIR-TADRUS, ETSIIAA, University of Valladolid, Palencia, Castile and León, 34004, Spain; 2 InTerACT (UP 2018.C102), UniLaSalle, Beauvais, Hauts-de-France, 60000, France; 3 Umeå School of Business, Economics and Statistics, Umeå University, Umeå, 901 87, Sweden; 4 Department of Agricultural Economics, School of Agriculture, Aristotle University of Thessaloniki, Thessaloniki, 54124, Greece; 5 CIDTFF, Departamento de Educação e Psicologia, Universidade de Aveiro, Aveiro, Aveiro District, 3810-193, Portugal; 6 Núcleo Interativo de Astronomia e Inovação em Educação, São Domingos de Rana, Lisbon, 2785-817, Portugal; 7 Facultad de Educación de Soria, Universidad de Valladolid, Soria, Castile and León, 42004, Spain; 8 Consorzio Scuola Comunità Impresa, Novara, Piedmont, 28100, Italy; 9 Reitoria da Universidade Nova de Lisboa, Campus de Campolide, Lisboa, 1099-085, Portugal

**Keywords:** Education for Sustainable Development, Open Badges, Assessment, Knowledge-Skills-Attitudes, Sustainability Competencies

## Abstract

**Background:**

The European sustainability competence framework (GreenComp) lacks clearly defined learning outcomes, hindering its effective implementation and assessment. This paper addresses this critical gap by developing measurable learning outcomes within the OpenPass4Climate project.

**Methods:**

A structured approach involving co-design among educational stakeholders, expert validation using a modified Delphi method, and comprehensive coverage analysis was employed. This iterative process led to the development of 40 learning outcomes aligned with GreenComp’s four competence areas. These outcomes were formulated to be observable, measurable, and assessable.

**Results:**

The process successfully yielded 40 learning outcomes, providing concrete, actionable targets that operationalize GreenComp’s theoretical framework into practical educational tools. This developed framework facilitates the recognition of sustainability competencies through Open Badges and supports the integration of sustainability education across formal, non-formal, and informal learning contexts.

**Conclusions:**

This work contributes to advancing Education for Sustainable Development. However, challenges persist in ensuring universal applicability, balancing standardization with contextual adaptation, and maintaining the holistic nature of sustainability within micro-credentialing systems.

## Abstract (fr)

**Contexte:**

Le cadre européen de compétences en matière de durabilité (GreenComp) manque de résultats d'apprentissage clairement définis, ce qui entrave sa mise en œuvre et son évaluation efficaces. Cet article comble cette lacune critique en développant des résultats d'apprentissage mesurables dans le cadre du projet OpenPass4Climate.

**Méthodes:**

Une approche structurée impliquant la co-conception entre les parties prenantes éducatives, la validation par des experts utilisant une méthode Delphi modifiée, et une analyse de couverture complète a été employée. Ce processus itératif a conduit au développement de 40 résultats d'apprentissage alignés sur les quatre domaines de compétences de GreenComp. Ces résultats ont été formulés pour être observables, mesurables et évaluables.

**Résultats:**

Le processus a permis de produire avec succès 40 résultats d'apprentissage, fournissant des objectifs concrets et réalisables qui opérationnalisent le cadre théorique de GreenComp en outils éducatifs pratiques. Ce cadre développé facilite la reconnaissance des compétences en durabilité grâce aux Open Badges et soutient l'intégration de l'éducation à la durabilité dans les contextes d'apprentissage formel, non formel et informel.

**Conclusions:**

Ce travail contribue à faire progresser l'Éducation au Développement Durable. Cependant, des défis persistent pour assurer l'applicabilité universelle, équilibrer la standardisation avec l'adaptation contextuelle, et maintenir la nature holistique de la durabilité au sein des systèmes de micro-certification.

## Abstract (it)

**Contesto:**

Il quadro europeo delle competenze sulla sostenibilità (GreenComp) manca di risultati di apprendimento chiaramente definiti, ostacolando la sua implementazione e valutazione efficaci. Questo articolo affronta questa lacuna critica sviluppando risultati di apprendimento misurabili all'interno del progetto OpenPass4Climate.

**Metodi:**

È stato impiegato un approccio strutturato che coinvolge la co-progettazione tra le parti interessate educative, la validazione degli esperti utilizzando un metodo Delphi modificato e un'analisi di copertura completa. Questo processo iterativo ha portato allo sviluppo di 40 risultati di apprendimento allineati con le quattro aree di competenza di GreenComp. Questi risultati sono stati formulati per essere osservabili, misurabili e valutabili.

**Risultati:**

Il processo ha prodotto con successo 40 risultati di apprendimento, fornendo obiettivi concreti e attuabili che operazionalizzano il quadro teorico di GreenComp in strumenti educativi pratici. Questo quadro sviluppato facilita il riconoscimento delle competenze sulla sostenibilità attraverso gli Open Badges e supporta l'integrazione dell'educazione alla sostenibilità nei contesti di apprendimento formale, non formale e informale.

**Conclusioni:**

Questo lavoro contribuisce al progresso dell'Educazione allo Sviluppo Sostenibile. Tuttavia, persistono sfide nel garantire l'applicabilità universale, bilanciare la standardizzazione con l'adattamento contestuale e mantenere la natura olistica della sostenibilità all'interno dei sistemi di micro-certificazione.

## Abstract (de)

**Hintergrund:**

Dem europäischen Nachhaltigkeitskompetenz-Rahmen (GreenComp) fehlen klar definierte Lernergebnisse, was seine effektive Umsetzung und Bewertung behindert. Dieser Artikel behandelt diese kritische Lücke durch die Entwicklung messbarer Lernergebnisse innerhalb des OpenPass4Climate-Projekts.

**Methoden:**

Ein strukturierter Ansatz mit Co-Design unter Bildungsakteuren, Expertenvalidierung mittels einer modifizierten Delphi-Methode und umfassender Abdeckungsanalyse wurde angewandt. Dieser iterative Prozess führte zur Entwicklung von 40 Lernergebnissen, die mit den vier Kompetenzbereichen von GreenComp übereinstimmen. Diese Ergebnisse wurden formuliert, um beobachtbar, messbar und bewertbar zu sein.

**Ergebnisse:**

Der Prozess erbrachte erfolgreich 40 Lernergebnisse und lieferte konkrete, umsetzbare Ziele, die GreenComps theoretischen Rahmen in praktische Bildungsinstrumente operationalisieren. Dieser entwickelte Rahmen erleichtert die Anerkennung von Nachhaltigkeitskompetenzen durch Open Badges und unterstützt die Integration von Nachhaltigkeitsbildung in formale, non-formale und informelle Lernkontexte.

**Schlussfolgerungen:**

Diese Arbeit trägt zur Weiterentwicklung der Bildung für nachhaltige Entwicklung bei. Dennoch bestehen Herausforderungen bei der Gewährleistung universeller Anwendbarkeit, der Ausbalancierung von Standardisierung mit kontextueller Anpassung und der Beibehaltung der ganzheitlichen Natur der Nachhaltigkeit innerhalb von Mikro-Zertifizierungssystemen.

## Abstract (es)

**Antecedentes:**

El marco europeo de competencias en sostenibilidad (GreenComp) carece de resultados de aprendizaje claramente definidos, lo que dificulta su implementación y evaluación efectivas. Este artículo aborda esta brecha crítica mediante el desarrollo de resultados de aprendizaje medibles dentro del proyecto OpenPass4Climate.

**Métodos:**

Se empleó un enfoque estructurado que involucra el co-diseño entre las partes interesadas educativas, la validación de expertos utilizando un método Delphi modificado y un análisis integral de cobertura. Este proceso iterativo condujo al desarrollo de 40 resultados de aprendizaje alineados con las cuatro áreas de competencia de GreenComp. Estos resultados fueron formulados para ser observables, medibles y evaluables.

**Resultados:**

El proceso produjo exitosamente 40 resultados de aprendizaje, proporcionando objetivos concretos y accionables que operacionalizan el marco teórico de GreenComp en herramientas educativas prácticas. Este marco desarrollado facilita el reconocimiento de competencias en sostenibilidad a través de Open Badges y apoya la integración de la educación en sostenibilidad en contextos de aprendizaje formal, no formal e informal.

**Conclusiones:**

Este trabajo contribuye al avance de la Educación para el Desarrollo Sostenible. Sin embargo, persisten desafíos para asegurar la aplicabilidad universal, equilibrar la estandarización con la adaptación contextual y mantener la naturaleza holística de la sostenibilidad dentro de los sistemas de micro-acreditación.

## Introduction

The mounting evidence of climate change and environmental degradation has catalyzed a fundamental shift in how educational institutions conceptualize their role in preparing learners for an uncertain future ( Nusche *et al*., 2024). The European Union’s response, embodied in the European sustainability competence framework (GreenComp) ( Bianchi *et al*., 2022), represents a significant policy intervention aimed at systematically developing citizens’ capabilities to navigate and address complex socio-ecological challenges. However, the translation of this ambitious framework into concrete educational practice remains problematic, revealing a critical gap between policy aspiration and pedagogical implementation.

This research is guided by a central methodological challenge: How can the GreenComp framework be operationalized into a set of valid, assessable learning outcomes that are fit for purpose within a European-level recognition system?

### EU competence frameworks for lifelong learning

The European Commission’s Joint Research Centre has developed a family of reference frameworks (LifeComp, EntreComp, DigComp, and GreenComp) to establish a shared understanding of key competences for citizens across formal, non-formal, and informal learning contexts. These “competency models” serve to describe learning outcomes and thus provide frameworks for operationalizing educational goals ( Klieme *et al*., 2004). However, they vary significantly in their level of detail and implementation guidance.

LifeComp, the European framework for “personal, social and learning to learn” competences, consists of nine competencies organized in three areas (Personal, Social, Learning to Learn). It is explicitly conceptual and non-prescriptive, intended to inform curriculum and learning-activity design, but does not include a progression model or a formal catalogue of learning outcomes.

EntreComp, the Entrepreneurship Competence Framework, articulates 15 competences in three interrelated areas (Ideas & Opportunities, Resources, Into Action), mapped along an 8-level progression model, and provides a comprehensive list of 442 learning outcomes to guide curriculum design and assessment ( Bacigalupo *et al*., 2016).

DigComp, the Digital Competence Framework for Citizens, defines 21 digital competences across five areas, described at eight proficiency levels, and is being extended via the “DigComp: Learning outcomes” project to develop intended learning outcomes and supporting materials for diverse application contexts. The development of learning outcomes for DigComp may serve as the basis for the European Digital Skills Certificate (EDSC), conceptualized as a quality label for digital skills certification across Europe.

GreenComp, the European Sustainability Competence Framework, identifies 12 sustainability competences organized into four areas (Embodying Sustainability Values, Embracing Complexity, Envisioning Sustainable Futures, Acting for Sustainability), each described by a competency descriptor plus Knowledge–Skills–Attitudes (KSAs). While the framework’s background document references “behavioral learning outcomes” ( Scalabrino, 2022), and the main framework document acknowledges the need to incorporate learning outcomes into sustainability pedagogies ( Bianchi *et al*., 2022), neither establishes formal, assessable learning outcomes nor a progression model, resulting in an “implementation vacuum”.

### The GreenComp framework – Focus of this study

EntreComp and DigComp already define or are in the process of defining detailed learning outcomes, and LifeComp remains at a conceptual level without specific outcome descriptors. In this study, the authors propose a set of measurable learning outcomes for GreenComp. In doing so, it aims to fill the current gap in operationalizing the sustainability competences of GreenComp, transforming its descriptive KSAs into assessable statements that educators and trainers can use to guide curriculum development, implementation, and evaluation.

### Current state of GreenComp assessment

The landscape of GreenComp assessment initiatives reveals a complex tapestry of approaches ( Javorka *et al*., 2024), each grappling with the fundamental challenge of operationalizing sustainability competencies into measurable outcomes. These diverse efforts, while demonstrating considerable innovation and commitment, collectively illuminate both the possibilities and persistent limitations in translating the framework's ambitious vision into practical assessment tools.

Traditional knowledge-focused assessment approaches dominate the current landscape, exemplified by TASK ^TM^ by Sulitest, which achieves comprehensive coverage of knowledge elements (100% alignment) while demonstrating more limited capacity in assessing skills (70% alignment) and attitudes (30% alignment) ( Sulitest, 2023). This imbalance, far from being merely technical, reflects deeper epistemological assumptions about what constitutes legitimate and measurable learning in sustainability education. CertiSkill ^TM^ similarly privileges cognitive dimensions through its certification exam based on 30 multiple-choice questions aligned with UNI/Pdr 109.2:2021 standards, while the Engineers4Europe project's Green Skills micro-credential, despite its innovative platform delivery and broad disciplinary appeal, ultimately relies on three knowledge-based quizzes to assess competencies in corporate sustainability and ESG.

Attempts to address the skills dimension of GreenComp have produced varied approaches with differing levels of rigor. The My Personality Skills ^®^ framework by Fundacja represents a private sector initiative offering the MY PS GreenComp Certificate, with examinations spanning all four GreenComp areas. However, the proprietary nature of this assessment raises questions about transparency and alignment with the open, collaborative spirit of the European competence frameworks. More innovative approaches emerge in the self-assessment domain, where BCN+B's Be Ready For Now employs a 48-question reflective instrument examining sustainability habits and knowledge, though its self-reporting methodology limits its utility for formal competence recognition.

The assessment of attitudes and values, arguably the most transformative dimension of sustainability education, remains particularly underdeveloped. The Advancing Sustainability Education's Sustainability Competence Mindset Map (SCMM) represents one of the few initiatives explicitly capturing attitudinal dimensions of sustainability competence capacity building. The SCMM is a formative self-reflective assessment based on the key sustainability competence frameworks ( Brundiers *et al*., 2021; Redman & Weik, 2021) and has been validated across disciplines in higher education ( Annelin & Boström, 2023; Annelin & Boström, 2024a; Annelin, 2025). It is a facilitating tool that supports teacher course planning and curriculum design, where teachers follow the progression of the affective intrapersonal attitudes of students over different periods of time (a course, a semester, a year, a program). Teachers can communicate the learning experience with the students ( Annelin, 2025) by using visual aids, reflective work, and complementary assessment techniques. The outcome of the SCMM assessment is automated via online tools integrated with data software to produce visual and comparable diagrams of the class progression. The tool is applied in the understanding that sustainability competence capacity building is a nonlinear, life-long learning process. This nonlinear relation is captured in the visual diagrams presented. The SCMM is used to complement the integration of knowledge and skills assessment necessary for a traditional holistic competence assessment.

Educational technology has offered novel pathways for engagement, though not necessarily for rigorous assessment. The GameComp–Green Edugames Erasmus+ project exemplifies this trend through its gamified approach, featuring 36 self-reflective questions that award badges and points, creating motivational affordances while formal competence certification calls for established validity and reliability standards.

Providing a more methodologically robust but more narrowly focused example, the EduCITY project uses mobile augmented reality games (MARGs) to assess sustainability competencies within smart learning city environments. This research-driven initiative demonstrates how a deep and specific assessment can be operationalized, but it also highlights the challenge of comprehensive coverage. Its assessment model, as presented, explicitly targets only one of the four GreenComp competence areas: ‘Embodying Sustainability Values’. Within this specific area, the project employs a rigorous mixed-methods approach, triangulating data from a validated GreenComp-based questionnaire with automated gameplay performance logs. While its scope is partial, the EduCITY model provides a structured and evidence-based method for evaluating competencies fostered through experiential, technology-enhanced learning, a level of detail not present in initiatives that claim broader coverage based on self-reflection or participation alone ( Marques *et al*., 2025).

The ASSESS project developed a more pedagogically sophisticated approach, creating rubrics for Green Competencies assessment across multiple levels (primary, secondary, higher education, and business contexts) with an accompanying mobile application. Their framework emphasizes dialogic assessment through co-creation of rubrics between teachers and students, peer and self-assessment capabilities, and collaborative discussion of evaluation criteria ( Sousa *et al*., 2024). While this dialogic approach is powerful for fostering student agency and formative development, its reliance on context-specific, co-created criteria stands in direct tension with the goals of a standardized recognition framework. The method's pedagogical strength in the classroom, therefore, presents a significant challenge for issuing portable credentials, whose currency depends on a high degree of comparability and inter-institutional trust.

The emergence of Open Badge initiatives represents a particularly promising yet problematic development in GreenComp operationalization. The GoGreen - Youth Navigator project's Green Badges system, hosted on the Cities of Learning platform and announced for pilot testing in January 2025, allows activity organizers to tag skills and competencies while enabling participants to earn badges and share learning experiences. However, the absence of defined learning outcomes or systematic assessment criteria reduces these badges to participation certificates rather than competence credentials. The same platform hosts the Climate for All project's “ Climate for All Facilitator Training”, where a 5-hour course includes a GreenComp Reflection badge recognizing "experiential understanding" gained through game-based learning, a formulation that sidesteps the challenge of defining what such understanding entails or how it might be evidenced beyond self-report.

More substantial efforts at badge-based recognition emerge in formal educational contexts. The University of Florence's "Education Towards Sustainable Futures" badge, launched in June 2023 as part of The European University for Well-Being [EUniWell] Erasmus+ project, recognizes completion of a hybrid course for future teachers comprising five days of training, self-learning activities, and evaluation. While this initiative demonstrates closer alignment with formal educational quality assurance through its explicit connection to the GreenComp methodological framework, it remains fundamentally a credential of participation rather than assessed competence.

This proliferation of initiatives collectively reveals both the widespread recognition of GreenComp's importance and the persistent conceptual and methodological challenges in its implementation. Each project's idiosyncratic interpretation of what constitutes evidence of sustainability competencies has created a fragmented landscape where innovation flourishes but coherence and comparability remain elusive. The absence of shared assessment criteria, quality assurance mechanisms, or interoperability frameworks means that a learner's sustainability competencies may be recognized entirely differently, or not at all, across institutional and national boundaries. This patchwork approach, while reflecting healthy experimentation, ultimately undermines GreenComp's vision of providing a common European framework for sustainability competencies, highlighting the urgent need for more systematic approaches to learning outcome definition and assessment methodology.

### The need for learning outcomes in GreenComp

Learning outcomes are essential for operationalizing competence frameworks, as they translate competencies into concrete, observable, and assessable statements of what learners should know and be able to do. The development of clear learning outcomes for GreenComp provides concrete targets for curriculum design and assessment in formal education, while guiding the development of non-formal learning experiences and workplace training. They enable the validation and recognition of informal learning through frameworks like Open Badges and support the evaluation of educational interventions for sustainability competence development.

Yet the challenge of developing learning outcomes for GreenComp extends beyond these technical considerations of measurement and validation. Sustainability competencies embody what Wals (2015) terms "gestalt competencies", i.e., holistic capabilities that emerge from the integration of knowledge, values, and action in ways that resist decomposition into discrete elements. This conceptualization reveals a fundamental paradox at the heart of our endeavor: educational systems require specificity for assessment and credentialing, while sustainability challenges demand integrated, systemic responses that transcend disciplinary and methodological boundaries.

The "wicked" nature of sustainability problems compounds this challenge. Characterized by complexity, uncertainty, value conflicts, and systemic interdependencies, these problems resist reduction to discrete, measurable outcomes. UNESCO's framework for Education for Sustainable Development ( UNESCO, 2019) emphasizes the need to develop three interconnected dimensions: cognitive (knowledge and understanding) ( Spaccatini *et al*., 2023), socio-emotional (values and attitudes) ( Lozano *et al*., 2022), and behavioral dimension (often referred to as conative, encompassing goal-directed actions and capabilities) ( Presseau *et al*., 2019). This tripartite model suggests that traditional approaches to learning outcome development, which typically privilege cognitive dimensions as more readily assessable, may be fundamentally insufficient for capturing the transformative potential of sustainability education.

Furthermore, sustainability education carries an explicitly normative dimension that distinguishes it from other competence frameworks in the European portfolio. While DigComp can maintain relative neutrality regarding digital tool use, treating technology as a means rather than an end, and EntreComp can embrace diverse entrepreneurial values within a broadly capitalist framework, GreenComp necessarily embeds particular values about human-nature relationships, intergenerational justice, and systemic transformation. This normative dimension creates profound tensions when developing learning outcomes that must be both prescriptive enough to guide learning and open enough to accommodate diverse cultural and ideological contexts.

The gestalt nature of sustainability competencies thus presents a double bind: decompose them too much and they lose their transformative essence, becoming mere technical skills divorced from the values and systemic thinking that give them meaning; leave them too holistic and they become impossible to assess, reducing credentials to vague attestations of exposure rather than evidence of capability. This tension between the holistic nature of sustainability competencies and the analytical requirements of educational systems represents not merely a technical challenge to be solved, but a fundamental feature of sustainability education that must be navigated thoughtfully.

Our approach to developing learning outcomes for GreenComp must therefore acknowledge and work within these tensions rather than attempting to resolve them prematurely. The outcomes must be specific enough to guide educational practice and enable recognition, while remaining open enough to accommodate the emergent, contextual, and transformative nature of sustainability learning. They must honor the gestalt quality of sustainability competencies while providing sufficient structure for implementation across diverse educational contexts. Most critically, they must maintain the normative vision of sustainability transformation while respecting cultural diversity and local knowledge systems.

### The OpenPass4Climate context

The OpenPass4Climate project (2022-1-FR01-KA220-HED-000089354) provides the institutional context for this learning outcome development, with its focus on creating an open recognition alliance system for sustainability competencies. The project aims to tackle climate change by implementing innovative teaching and learning methods, with one of its key objectives being the development of Open Badges for recognition of non-formal and informal learning related to sustainability competencies, ultimately leading to certification of competencies through Europass. This technological and institutional infrastructure shapes the requirements for learning outcomes, which must be granular enough for badge-based recognition while comprehensive enough to represent meaningful competency development. This presents a central methodological challenge: leveraging the accessibility of micro-credentials without succumbing to the very reductionism that a holistic framework like GreenComp seeks to avoid. The methodology described below was designed explicitly to navigate this tension.

## Methodology

The development of learning outcomes for GreenComp required a methodological approach that could navigate multiple tensions: between theoretical comprehensiveness and practical applicability, between standardization and contextual flexibility, and between expert knowledge and stakeholder accessibility. A systematic, iterative approach was chosen, comprising four interconnected phases: initial co-design, first expert validation, coverage analysis, and second expert validation. This multi-phase approach allowed for progressive refinement while maintaining alignment with both the GreenComp framework and CEDEFOP guidelines for learning outcome development.

### Phase 1: Initial co-design process

The co-design phase represented a deliberate departure from expert-driven approaches to learning outcome development. Rather than beginning with technical specifications derived from learning theory, we initiated a collaborative process involving stakeholders from the OpenPass4Climate consortium (CSCI Novara, NOVA University, UniLaSalle, and Valladolid University), including educators from formal higher education contexts, trainers from non-formal education settings, and learners representing diverse disciplinary backgrounds. This heterogeneous composition was essential for ensuring that the resulting learning outcomes would be meaningful across the varied contexts in which GreenComp might be implemented.

The co-design process unfolded through a series of structured workshops conducted over three months. Each workshop focused on a specific GreenComp competence area, with participants working in interdisciplinary groups to translate abstract KSA statements into concrete learning outcomes.

To structure the translation process, we employed Bloom’s revised taxonomy ( Anderson & Krathwohl, 2001) as a heuristic device rather than a rigid framework. Participants were encouraged to consider how each competence might manifest at different cognitive levels, from basic comprehension to creative synthesis. However, we found that many sustainability competencies resisted easy categorization within Bloom’s framework, particularly those involving affective dimensions or systems thinking. This led to iterative refinement of outcomes that attempted to capture the holistic nature of competencies while maintaining sufficient specificity for assessment purposes.

The initial co-design phase produced 36 learning outcomes, three for each of the 12 GreenComp competencies. These were formulated according to the Centre Européen pour le Développement de la Formation Professionnelle (CEDEFOP) guidelines for defining, writing and applying learning outcomes ( CEDEFOP, 2022), ensuring they were observable (describing a behavior or product that can be observed), measurable (assessable using specific criteria), and student-centered (focusing on what the learner will be able to do). Each learning outcome was formulated to integrate relevant knowledge, skills, and attitudes from the GreenComp framework, translating the abstract KSAs into concrete statements of what learners should demonstrate.

### Phase 2: First expert validation round

The expert validation phase employed a modified Delphi approach designed to balance quality assurance with practical constraints, such as the project timeline and expert availability. Our expert panel comprised 12 specialists selected based on demonstrated expertise in sustainability education, familiarity with European competence frameworks (evidenced through publications or project participation), and representation of diverse educational contexts (formal, non-formal, and informal settings).

The institutional diversity of the panel was carefully cultivated, with experts representing universities, vocational education institutions, and NGOs focused on sustainability education. This diversity proved crucial during the validation process, as experts brought culturally and contextually specific insights that challenged the universalist assumptions embedded in our initial formulations. The panel included representatives from various EU sustainability projects (such as TWIN-IN, ASSESS, EduC3, GrowLearnOut, GreenScent, GreenAtYou, and Entrepreneurship4All) and authors of GreenComp-related publications and initiatives.

The validation instrument consisted of a structured evaluation form for each learning outcome, incorporating both binary validity judgments and qualitative feedback. Experts evaluated each outcome against four criteria:

– Alignment: Clear relationship to the target GreenComp competence
– Measurability: Use of clear, assessable verbs and specific performance indicators
– Student-centricity: Focus on learner capabilities rather than teaching activities
– Observability: Potential for demonstration through observable behaviors or products

For each criterion, experts indicated whether the learning outcome was valid or not valid, accompanied by open-ended comments for improvement suggestions. A learning outcome was considered to require revision if any expert marked it as not valid on any criterion or if substantial concerns were raised in the qualitative feedback. The validation was conducted asynchronously over four weeks, allowing experts sufficient time for thoughtful evaluation.

Binary validation data from expert ratings were analyzed to identify outcomes requiring revision (those marked as not valid by any expert on any criterion). Learning outcomes were categorized as: (1) approved without changes if all experts marked them as valid across all criteria, or (2) requiring revision if one or more experts identified validity concerns. Qualitative feedback was analyzed thematically to identify recurring concerns and suggestions for improvement, with particular attention to cases where experts disagreed on validity judgments.

### Phase 3: Coverage analysis

Following the first validation round, we also conducted a systematic coverage analysis to assess how comprehensively the learning outcomes addressed all aspects of the GreenComp framework. This analysis employed a multi-dimensional mapping approach designed to identify potential gaps or imbalances in coverage.

The coverage analysis methodology involved three steps:

– Step 1: Matrix development. We created a comprehensive matrix mapping each validated learning outcome to specific knowledge, skill, and attitude statements within the GreenComp framework. Each cell in the matrix indicated whether a particular KSA element was addressed by one or more learning outcomes.
– Step 2: Gap identification. Through systematic analysis of the mapping matrix, we identified KSA elements that were either not addressed or insufficiently represented in the current outcome set. This analysis examined both horizontal coverage (across all competencies) and vertical coverage (across knowledge, skills, and attitudes within each competence).
– Step 3: Pattern analysis. We analyzed the patterns of gaps to understand whether they reflected systematic biases or specific challenges in operationalizing certain aspects of sustainability competencies. This involved examining whether gaps clustered around particular types of content (e.g., philosophical dimensions, cultural aspects, systemic perspectives).

The coverage analysis was conducted independently by three researchers, with discrepancies resolved through discussion and reference to the original GreenComp documentation. This triangulation approach enhanced the reliability of gap identification.

### Phase 4: Second expert validation round

Based on the experts’ first validation round feedback, revisions were made to the learning outcomes to improve their clarity, specificity, and evaluability. Concerning the coverage analysis findings, we developed strategies to address identified gaps through two approaches: (1) modification of existing learning outcomes to incorporate additional KSA elements, and (2) creation of new learning outcomes targeting unaddressed areas. All proposed changes were subjected to a second round of expert validation.

The second validation round followed a more focused protocol, presenting experts with:

– Detailed rationales for each proposed modification or addition
– Explicit mapping of how changes addressed prior comments or coverage gaps
– Comparison between the original and revised formulations

This round particularly focused on contentious additions, such as learning outcomes challenging anthropocentric perspectives or addressing resource conflict dynamics. Experts were asked to evaluate not only the technical quality of these outcomes but also their appropriateness within the GreenComp framework and potential implementation challenges.

## Results

The iterative development process produced a comprehensive set of 40 learning outcomes that operationalize the GreenComp framework while addressing critical gaps identified through systematic analysis. These results reflect the integration of stakeholder co-design, expert validation, and coverage enhancement across two major iterations.

### First iteration outcomes

The initial co-design phase successfully generated 36 learning outcomes, three for each of the 12 GreenComp competencies. The first expert validation round revealed strong overall alignment with the framework, with 21 outcomes (58.3%) approved without modifications and 15 outcomes (41.7%) requiring revisions.

The outcomes receiving unanimous or near-unanimous approval clustered in areas where GreenComp provides clear, concrete guidance. These included:

– Area I (Embodying Sustainability Values): 3 outcomes addressing critique of sustainability arguments, inclusive solution construction, and nature preservation advocacy.
– Area II (Embracing Complexity): 8 outcomes focusing on systems analysis, interaction mapping, critical evaluation, and problem differentiation.
– Area III (Envisioning Sustainable Futures): 2 outcomes addressing decision-making with tradeoffs and interdisciplinary synthesis.
– Area IV (Acting for Sustainability): 8 outcomes covering political analysis, advocacy, coalition building, and initiative evaluation.

Concerning the remaining 15 learning outcomes, revisions included changes only to components for 4 learning outcomes (11.1%) and changes to both the main statement and other components for 11 learning outcomes (30.6%). Looking at component changes, observable components were revised in 14 learning outcomes (93.3% of changed learning outcomes), measurable components in 10 learning outcomes (66.7%), and evaluation methods in 12 learning outcomes (80.0%).

The revisions centered around several key themes:

– Improved action verbs: Many learning outcomes were revised to use more precise action verbs that clearly signal the level of learning expected. For example, "integrate" was changed to "connect," "demonstrate" was changed to "adapt," and "develop" was changed to "formulate".
– Learner-centered approach: Observable components were revised to focus explicitly on what the learner can do rather than abstract qualities or outputs. For example, "Integration observable through synthesis of individual contributions" was changed to "The learner can map their personal sustainability actions to specific elements of shared sustainability frameworks, identify roles within collective initiatives, develop action plans..."
– Increased specificity and context: Learning outcomes were revised to provide clearer contexts and specify the scope of application. For example, "Implement principles of resource efficiency, reuse, and sharing" was changed to "Apply resource efficiency, reuse, and sharing principles in personal and group contexts."
– Enhanced measurability: Vague measurable components were replaced with more concrete, assessable criteria. For example, "Scoring coherence and collective impact of coordinated actions" was changed to "Rubrics can assess the coherence between individual and collective actions, clarity of role definition, evidence of adaptation to collective contexts, and tangible contributions to shared sustainability goals."
– Accessibility for non-specialists: Learning outcomes that required specialist knowledge were revised to be more accessible. For example, evaluation methods like "Environmental impact assessment projects" were changed to "Guided environmental impact assessment projects" to make them more accessible to non-specialists.
– Balancing individual and collaborative elements: Many learning outcomes were revised to balance individual action with collaborative and social dimensions. For example, "Initiate precautionary actions" was changed to "Apply the precautionary principle through targeted actions", focusing on application rather than leadership/initiation, which might be intimidating for some learners.
– Clarity in complex concepts: Ambiguous or complex concepts were clarified. For example, "Develop strategies to manage transitions from environmental changes" was changed to "Formulate adaptive strategies to manage socio-ecological transitions necessitated by environmental and societal changes," providing clearer parameters for what constitutes a transition.

### Coverage analysis findings

The systematic coverage analysis revealed significant patterns in how the initial 36 learning outcomes addressed the GreenComp framework:

Well-covered areas included knowledge elements (87% of GreenComp knowledge statements were addressed), systems thinking aspects (with a comprehensive coverage across all three competence areas), and action-oriented skills (with a strong representation in Area IV, Acting for Sustainability).

With regard to the identified gaps, the analysis revealed systematic underrepresentation in four critical areas:

– Philosophical and ethical dimensions, with limited coverage of the intrinsic value of nature, ecocentric perspectives, and environmental ethics foundations.
– Cultural diversity in sustainability, with insufficient attention to diverse cultural conceptualizations of human-nature relationships.
– Economic transformation, with the absence of learning outcomes addressing alternative economic models and decoupling strategies.
– Conflict and resource dynamics, with no outcomes explicitly connecting resource depletion to social conflicts and environmental disasters.

Concerning coverage imbalances, attitudes were less comprehensively addressed (62% coverage) compared to knowledge (87%) and skills (79%); Area I (Embodying Sustainability Values) showed the most significant gaps, particularly in competence 1.3 (Promoting Nature); and systemic and structural dimensions were underrepresented relative to individual behavioral changes.

### Enhancement strategies

To address identified gaps while maintaining a manageable number of learning outcomes, we employed two strategies:

The first strategy entailed the modification of existing learning outcomes. Three existing learning outcomes were expanded to incorporate additional KSA elements:

– Valuing sustainability learning outcome 1: Added cultural and generational dimensions to address the gap in coverage of 1.1-K4.
– Supporting fairness learning outcome 1: Integrated personal needs assessment with resource minimization to address the gap in coverage of 1.2-S2.
– Supporting fairness learning outcome 2: Included varying capacities for sustainability promotion to address the gap in coverage of 1.2-K4.

The second strategy involved the creation of new learning outcomes. Four new learning outcomes were developed to address critical gaps in the KSAs of the associated GreenComp competencies:

– Analyzing consumption drivers and their environmental and social impacts (1.1-K5).
– Explaining models for decoupling economic activity from resource consumption (1.3-K6).
– Analyzing how resource depletion contributes to social conflicts and environmental disasters (1.3-K5).
– Challenging anthropocentric perspectives in environmental protection approaches (1.3-A2).

### Second iteration results

The second expert validation round focused on the eight modified or new learning outcomes. Three learning outcomes required further refinement based on expert feedback:

– Valuing Sustainability learning outcome 4 was enhanced to explicitly include social alongside environmental sustainability dimensions, with corresponding adjustments to measurable criteria and evaluation methods.
– Promoting Nature learning outcome 5 was expanded to balance coverage between social conflicts and environmental disasters, addressing expert concerns about incomplete scope. Measurable criteria were changed to explicitly include “connections between resource depletion and environmental disasters” and “feedback loops between resource scarcity and environmental degradation.”
– Promoting Nature learning outcome 6: The suggestion to include “attributing equal importance to human and non-human beings” was carefully considered but ultimately refined to emphasize that approaches should consider multiple species’ interests “as morally significant in their own right,” maintaining philosophical accuracy while avoiding absolute equality claims that might not align with established ecocentric positions.

### Final learning outcome distribution

The development process resulted in 40 learning outcomes ( Table 1, Annex I in the Extended Data) distributed across the four GreenComp competence areas with a strategic emphasis on foundational values. Area I: Sustainability Values contains 13 learning outcomes, reflecting its foundational importance for sustainability competence development as the ethical grounding that informs all other aspects. The remaining three areas (Area II: Embracing Complexity, Area III: Envisioning Sustainable Futures, and Area IV: Acting for Sustainability) each contain 9 learning outcomes, providing balanced coverage of the analytical, future-oriented, and action-focused dimensions of sustainability competence.

**Table 1.** Complete set of forty GreenComp learning outcomes.

Competence area	Competence	Learning outcome	KSA coverage
I. Sustainability values	*1.1 Valuing* * sustainability*	1. Critique sustainability arguments, policies, and actions by analyzing their underlying values and principles, including cultural and generational differences	1.1-K1, 1.1-K2, 1.1-K4, 1.1-S1, 1.1-S2, 1.1-A3, 1.1-A4
		2. Negotiate solutions that reach a consensus aligned with specific sustainability principles while respecting diverse stakeholder perspectives	1.1-S4, 1.1-S5, 1.1-A2
		3. Design and implement a personal action plan applying concrete sustainability principles to daily choices	1.1-K3, 1.1-K6, 1.1-S3, 1.1-A1
		4. Analyze how consumption drivers impact resource demand and environmental and social sustainability	1.1-K5, 1.1-S2
	*1.2 Supporting* * fairness*	1. Apply concepts of equity, environmental justice, and intergenerational fairness when addressing various sustainability challenges while questioning personal and societal resource needs	1.2-K1, 1.2-K2, 1.2-S1, 1.2-S2, 1.2-A1
		2. Construct inclusive solutions that respect diverse cultural views and address varying capacities to promote sustainability	1.2-K4, 1.2-S3, 1.2-S4
		3. Advocate for the preservation of nature for current and future generations	1.2-K3, 1.2-A2, 1.2-A3
	*1.3 Promoting* * nature*	1. Explain the interconnectedness between human well- being and ecosystem health, including analyzing personal impacts on natural systems	1.3-K1, 1.3-K2, 1.3-K3, 1.3-S1, 1.3-S5, 1.3-A4
		2. Participate actively in specific practices that restore, regenerate, and promote harmonious coexistence with nature	1.3-S4, 1.3-A1, 1.3-S3, 1.3-A5
		3. Value the rights and roles of other life forms in maintaining ecological balance	1.3-K4, 1.3-S2, 1.3-A3
		4. Explain models for decoupling economic activity from resource consumption	1.3-K6
		5. Analyze how resource depletion contributes to social conflicts and environmental disasters	1.3-K5
		6. Challenge anthropocentric perspectives in approaches to environmental protection	1.3-A2
II. Embracing complexity	*2.1 Systems* * thinking*	1. Analyze how human activities across domains impact environmental and societal systems	2.1-K1, 2.1-K2, 2.1-S1, 2.1-S2, 2.1-A1, 2.1-A3
		2. Map the interactions, feedback, and cascading effects within complex sustainability issues	2.1-K4, 2.1-K5, 2.1-S3, 2.1-S5, 2.1-A2, 2.1-A4, 2.1-A5
		3. Apply systems modeling, life cycle assessment techniques, and resource minimization methods to sustainability challenges	2.1-K3, 2.1-S4
	*2.2 Critical* * thinking*	1. Evaluate the reliability of sustainability information sources and claims	2.2-K1, 2.2-K4, 2.2-S3, 2.2-S5, 2.2-A5
		2. Deconstruct arguments to identify underlying assumptions, biases, and contexts	2.2-K2, 2.2-K3, 2.2-K5, 2.2-S2, 2.2-A2
		3. Formulate evidence-based perspectives on sustainability issues that integrate diverse human and non-human considerations	2.2-K1, 2.2-S1, 2.2-S4, 2.2-A1, 2.2-A3, 2.2-A4
	*2.3 Problem * *framing*	1. Differentiate between simple, complicated, and complex sustainability problems	2.3-K1, 2.3-K2, 2.3-K4, 2.3-A2
		2. Transform sustainability problem definitions by systematically integrating diverse stakeholder viewpoints and systems-level considerations	2.3-K3, 2.3-S1, 2.3-S2, 2.3-A4
		3. Select appropriate strategies to mitigate, adapt, and solve sustainability challenges	2.3-K5, 2.3-S3, 2.3-S4, 2.3-S5
III. Envisioning sustainable futures	*3.1 Futures literacy*	1. Create alternative scenario models that question current paradigms and envision transformative sustainable futures	3.1-K1, 3.1-K5, 3.1-S1, 3.1-A4
		2. Assess opportunities, risks, and implications of different future scenarios	3.1-K4, 3.1-S2, 3.1-A3
		3. Design a pathway with interventions toward a preferred sustainable future	3.1-K2, 3.1-K3, 3.1-S3, 3.1-S4, 3.1-A1, 3.1-A2
	*3.2 Adaptability*	1. Adapt personal practices in response to changing sustainability contexts, constraints, and new information	3.2-K4, 3.2-S1, 3.2-S2, 3.2-A2, 3.2-A4
		2. Make decisions by evaluating sustainability tradeoffs across domains and scales	3.2-K5, 3.2-S3, 3.2-A5
		3. Formulate adaptive strategies to manage socio-ecological transitions necessitated by environmental and societal changes	3.2-K1, 3.2-K2, 3.2-K3, 3.2-S3, 3.2-S4, 3.2-A1, 3.2-A3
	*3.3 Exploratory* * thinking*	1. Synthesize knowledge from various disciplines to approach sustainability issues	3.3-K1, 3.3-K4, 3.3-S1, 3.3-S2, 3.3-S3
		2. Experiment with innovative problem-solving methods, such as systems thinking, to address sustainability challenges	3.3-K2, 3.3-K3, 3.3-A1, 3.3-A4
		3. Accommodate diverse perspectives when exploring sustainable solutions	3.3-S5, 3.3-A2, 3.3-A3
IV. Acting for sustainability	*4.1 Political* * agency*	1. Analyze how political systems, policies, and power impact sustainability	4.1-K1, 4.1-S1, 4.1-A4
		2. Apply knowledge and skills for effective participation in democratic processes to advance sustainability policies	4.1-K3, 4.1-S2, 4.1-S4, 4.1-A1
		3. Advocate for political responsibility and sustainability accountability	4.1-K2, 4.1-K4, 4.1-S3, 4.1-A2, 4.1-A3
	*4.2 Individual* * initiative*	1. Evaluate one’s potential to drive positive environmental changes	4.2-K1, 4.2-K3, 4.2-K4, 4.2-K5, 4.2-S4, 4.2-S6, 4.2-A2, 4.2-A3, 4.2-A4,
		2. Apply resource efficiency, reuse, and sharing principles in personal and group contexts	4.2-S1, 4.2-S5, 4.2-A5
		3. Apply the precautionary principle through targeted actions that help prevent ecological and human harm	4.2-K2, 4.2-S2, 4.2-S3, 4.2-A1
	*4.3 Collective* * action*	1. Build diverse coalitions by identifying stakeholder strengths and roles	4.3-K1, 4.3-S1, 4.3-S5
		2. Facilitate, initiate, or actively participate in inclusive community processes for coordinated sustainability action	4.3-K2, 4.3-K3, 4.3-K4, 4.3-S2, 4.3-S3, 4.3-S4, 4.3-A2, 4.3-A5
		3. Connect personal sustainability actions to the collective implementation of explicitly defined sustainability visions	4.3-S6, 4.3-A1, 4.3-A3, 4.3-A4

## Discussion

### Situating our contribution within the GreenComp assessment landscape

The proliferation of assessment initiatives targeting GreenComp competencies reveals both the recognized importance of sustainability education and the persistent challenges in its operationalization. Our review of existing approaches reveals a fragmented landscape characterized by partial coverage, methodological limitations, and implicit assumptions about the nature of sustainability competencies.

The dominant pattern across existing initiatives is the privileging of cognitive dimensions over affective and behavioral ones. TASK™ by Sulitest, despite its comprehensive alignment with GreenComp’s knowledge elements (100% coverage), achieves only 70% coverage of skills and 30% of attitudes. This imbalance reflects not merely technical limitations but deeper assumptions about what constitutes legitimate educational assessment. The measurability of factual knowledge through traditional testing formats creates path dependencies that marginalize the transformative dimensions of sustainability education.

This limitation is critical because, as Mezirow (1981) argues, a core aim of education for sustainability must be to transform learners' perspectives, helping them reconsider and change problematic frames of reference while reshaping fundamental attitudes. Our contribution responds directly to this transformative imperative. Unlike initiatives that primarily focus on knowledge assessment, the developed learning outcomes address all three dimensions of learning (cognitive, social-emotional, and behavioral) across all 12 GreenComp competencies. In contrast to self-assessment tools, the learning outcomes are designed to be observable through specific behaviors or products and measurable using defined criteria. Rather than treating knowledge, skills, and attitudes as separate elements, the learning outcomes integrate these components into holistic statements of what learners should be able to demonstrate.

The philosophical grounding of our approach also distinguishes it from technocratic assessment initiatives. By explicitly incorporating learning outcomes that challenge anthropocentric worldviews and require engagement with environmental ethics, we acknowledge that sustainability education cannot be value-neutral. This normative dimension, while complicating assessment, is essential for the transformative potential of sustainability education.

### Challenges and limitations

The development of learning outcomes for GreenComp revealed several persistent challenges that reflect deeper tensions in sustainability education. The most fundamental challenge concerns the tension between the holistic, transformative nature of sustainability competencies and the analytical requirements of learning outcome specifications. The learning outcomes are presented as discrete units, but sustainability competencies, with their emphasis on systems thinking, value transformation, and collective action, resist decomposition into discrete, observable behaviors.

Cultural and contextual variability presents another significant challenge. While our expert panel represented diverse European contexts, the learning outcomes inevitably embed certain cultural assumptions about sustainability, education, and assessment. What constitutes “supporting fairness” or “promoting nature” varies significantly across cultural contexts, and our formulations, despite attempts at inclusivity, reflect predominantly Western European perspectives. Future research should therefore focus on validating and adapting these learning outcomes in non-European contexts, engaging with diverse cultural knowledge systems to enrich and decolonize the framework.

The assessment challenge remains particularly acute. While we have specified observable and measurable dimensions for each learning outcome, the actual assessment of complex competencies like “challenging anthropocentric perspectives” requires sophisticated approaches that go beyond traditional testing. Portfolio assessment, peer evaluation, and community-based projects offer possibilities, but these require resources and expertise that may not be available in all educational contexts.

### Implications for Practice

The implementation of these learning outcomes in educational practice requires consideration of context, resources, and pedagogical approaches. Educators implementing these outcomes should view them not as rigid prescriptions but as flexible frameworks that can be adapted to local contexts while maintaining alignment with GreenComp’s core intentions.

Digital badge systems offer several affordances for competency recognition. Badges provide granular documentation of specific competencies achieved ( Hickey & Willis, 2017). They can increase learner motivation through visible progress markers and social recognition ( Abramovich *et al*., 2013). However, implementation approaches vary significantly in their effectiveness. Some badge systems risk fragmenting holistic competencies into disconnected micro-credentials, potentially undermining the integrated nature of sustainability education ( Lockley *et al*., 2016). This fragmentation echoes a broader challenge in sustainability education: the tendency toward isolated learning experiences that fail to connect across disciplines, contexts, and real-world applications ( Christie *et al*., 2013; Sipos *et al*., 2008). Students consistently report frustration with compartmentalized sustainability courses that don't translate into integrated understanding or action ( Hopkinson & James, 2010). More effective badge implementations recognize 'constellations' of learning outcomes, requiring learners to demonstrate competence across cognitive, behavioral, and affective domains. For instance, a badge might require combining a systems analysis with a collective action initiative before a credential is awarded. This approach better honors the holistic 'gestalt' nature of sustainability competence. However, the labor market reception of such credentials remains mixed, with some employers valuing the specific competency documentation while others remain unfamiliar with badge-based credentials ( Carey & Stefaniak, 2018; Raish & Rimland, 2016).

Professional development for educators represents perhaps the most critical factor for the successful implementation of these learning outcomes. The competencies assume educators who can facilitate complex learning processes, assess transformative capabilities, and navigate the inherently value-laden terrain of sustainability education ( Annelin & Boström, 2024b). This extends far beyond technical training in assessment methods. Educators require deep engagement with sustainability concepts, systems thinking pedagogies, and transformative learning approaches ( Burns, 2015; Sterling, 2024). They need support in developing facilitation skills for managing potentially contentious discussions about values, justice, and systemic change. Furthermore, educators themselves must engage in the same transformative learning processes they seek to facilitate, examining their own assumptions about human-nature relationships and sustainability transitions ( Moore, 2005). Without substantial investment in such comprehensive professional development, even the most carefully designed learning outcomes risk being implemented in ways that perpetuate transmissive rather than transformative education.

### Future directions

The development of these learning outcomes for GreenComp represents a beginning rather than an endpoint. Future work should focus on several critical areas to ensure their effective and equitable implementation: (1) extensive field-testing across diverse educational contexts, (2) development of robust assessment frameworks and tools, (3) creation of comprehensive implementation guides and supporting materials, (4) establishment of progression pathways for competency development over time, and (5) integration with existing curriculum frameworks and qualification systems.

A primary step is to conduct extensive field testing in diverse educational settings. This is essential not only to validate the practical utility of the learning outcomes but also to inform the development of guidance for their contextual adaptation. To ensure the framework's long-term relevance as sustainability challenges evolve, it would be prudent to establish a review cycle to periodically reassess its effectiveness.

The development of robust assessment frameworks is another priority. This includes creating tools and rubrics that can capture the complexity of sustainability competencies while remaining practical for educators. Crucially, this work should include examples of low-resource assessment methods to ensure applicability across varied economic and institutional contexts.

To support educators, the creation of comprehensive implementation guides is vital. Such guides could be complemented by integrated learning modules that address multiple competencies simultaneously and supporting materials that address prerequisite knowledge requirements. Building on this, the creation of progression pathways showing how learners can develop competencies over time would address a significant gap and would help educators design coherent learning sequences. While the current framework avoids specifying rigid levels to maintain flexibility, future iterations could consider creating differentiated versions for different educational levels (e.g., primary, secondary, vocational, higher education) to enhance usability.

Finally, integration with existing curriculum frameworks and qualification systems presents both an opportunity and a challenge. The learning outcomes must be translated into language and structures that align with national and regional education systems while maintaining their transformative potential. This requires ongoing dialogue between European-level framework development and national implementation efforts.

## Conclusions

This paper has presented the methodology and results of developing learning outcomes for the European sustainability competence framework (GreenComp) as part of the OpenPass4Climate project. The development process, involving co-design among educational stakeholders, validation by an international panel of experts, and comprehensive coverage analysis, has produced 40 learning outcomes that operationalize GreenComp’s competencies while addressing critical gaps in the original framework.

The resulting learning outcomes represent more than technical specifications for assessment. They embody a vision of sustainability education that integrates cognitive understanding, emotional engagement, and behavioral transformation. By explicitly incorporating philosophical dimensions, challenging anthropocentric assumptions, and connecting individual and collective action, they point toward a more transformative approach to sustainability education than purely skills-based frameworks allow.

The tensions revealed in this process (between standardization and contextualization, between measurability and transformation, between individual and systemic change) are not merely technical challenges to be resolved but fundamental features of sustainability education. The learning outcomes we have developed attempt to hold these tensions productively rather than resolve them prematurely.

As the climate crisis accelerates and planetary boundaries strain, the urgency of sustainability education intensifies. Our learning outcomes provide infrastructure for this educational transformation, but infrastructure alone cannot create change. The transformation requires educators willing to challenge disciplinary boundaries, institutions ready to restructure assessment paradigms, and societies prepared to question fundamental values. The learning outcomes are tools for this work, but the work itself remains ahead.

The ultimate test of our framework will not be its technical elegance or theoretical sophistication but its capacity to catalyze meaningful change. Do learners who develop these competencies act differently in the world? Do educational programs using these outcomes produce graduates capable of systemic thinking and collective action? Does the framework contribute to the broader transformation toward sustainability? These questions cannot be answered through methodology but only through practice, experimentation, and ongoing reflection.

In offering these learning outcomes to the educational community, we invite not just implementation but co-evolution. The framework should grow, adapt, and transform through use. It should incorporate perspectives excluded from our initial process. It should respond to emerging sustainability challenges and educational innovations. Most fundamentally, it should serve not as an endpoint but as a catalyst for the ongoing work of creating educational systems adequate to our planetary moment.

## Ethics and consent

Ethical approval and consent were not required.

## Data Availability

UVaDOC Documentary Repository of the University of Valladolid: Operationalizing the European sustainability competence framework: Development and validation of learning outcomes for GreenComp. https://uvadoc.uva.es/handle/10324/76293 This project contains the following extended data: – Annex I: GreenComp learning outcomes. All data are available under the terms of the Creative Commons Zero "No rights reserved" data waiver (CC0 Public domain dedication).

## References

[ref-1] Abramovich S Schunn C Higashi RM : Are badges useful in education? It depends upon the type of badge and expertise of learner. *Educ Technol Res Dev.* 2013;61(2):217–232. 10.1007/s11423-013-9289-2

[ref-2] Anderson LW Krathwohl DR : A taxonomy for learning, teaching, and assessing: a revision of Bloom’s taxonomy of educational objectives: complete edition.Addison Wesley Longman,2001. Reference Source

[ref-3] Annelin A : Sustainability competence mindset: building capacity with students. *Int J Sustain High Educ.* (forthcoming; DOI not provided),2025.

[ref-4] Annelin A Boström GO : An assessment of key sustainability competencies: a review of scales and propositions for validation. *Int J Sustain High Educ.* 2023;24(9):53–69. 10.1108/ijshe-05-2022-0166

[ref-5] Annelin A Boström GO : Sustainability competence assessment validation.In: T. Wall, L. Viera Trevisan, W. Leal Filho, & A. Shore (Eds.), *Sustainability in business education, research and practices.*Springer Nature Switzerland,2024a;209–226. 10.1007/978-3-031-55996-9_14

[ref-6] Annelin A Boström GO : Interdisciplinary perspectives on sustainability in higher education: a sustainability competence support model. *Front Sustain.* 2024b;5: 1416498. 10.3389/frsus.2024.1416498

[ref-7] Bacigalupo M Kampylis P Punie Y : EntreComp: the entrepreneurship competence framework.Publication Office of the European Union,2016. 10.2791/593884

[ref-8] Bianchi G Pisiotis U Cabrera Giraldez M : GreenComp - The European sustainability competence framework.Publications Office of the European Union,2022. 10.2760/13286

[ref-9] Brundiers K Barth M Cebrián G : Key competencies in sustainability in higher education—Toward an agreed-upon reference framework. *Sustain Sci.* 2021;16(1):13–29. 10.1007/s11625-020-00838-2

[ref-32] Burns H : Transformative sustainability pedagogy: learning from ecological systems and indigenous wisdom. *J Transform Educ.* 2015;13(3):259–276. 10.1177/1541344615584683

[ref-11] Carey KL Stefaniak JE : An exploration of the utility of digital badging in higher education settings. *Education Tech Research Dev.* 2018;66(5):1211–1229. 10.1007/s11423-018-9602-1

[ref-10] CEDEFOP: Defining, writing and applying learning outcomes: a European handbook (2nd ed.).Publications Office,2022. 10.2801/703079

[ref-12] Christie BA Miller KK Cooke R : Environmental sustainability in higher education: how do academics teach? *Environ Educ Res.* 2013;19(3):385–414. 10.1080/13504622.2012.698598

[ref-13] Hickey DT Willis JE : Where open badges appear to work better: findings from the Design Principles Documentation Project.Center for Research on Learning and Technology, Indiana University,2017. Reference Source

[ref-14] Hopkinson P James P : Practical pedagogy for embedding ESD in science, technology, engineering, and mathematics curricula. *Int J Sustain High Educ.* 2010;11(4):365–379. 10.1108/14676371011077586

[ref-15] Javorka Z Nieth L Marinelli E : GreenComp in practice: case studies on the use of the European competence framework—Analytical report.Publications Office of the European Union,2024. 10.2766/053738

[ref-16] Klieme E Avenarius H Blum W : The development of national educational standards: an expertise. Bundesministerium für Bildung und Forschung,2004. Reference Source

[ref-17] Lockley J Derryberry A West D : Drivers, affordances, and challenges of digital badges. In: D. Ifenthaler, N. Bellin Mularski, & D. K. Mah (Eds.), *Foundation of digital badges and micro credentials*. Springer,2016;55–70. 10.1007/978-3-319-15425-1_4

[ref-18] Lozano A López R Pereira FJ : Impact of Cooperative Learning and Project-Based Learning through emotional intelligence: a comparison of methodologies for implementing SDGs. *Int J Environ Res Public Health.* 2022;19(24): 16977. 10.3390/ijerph192416977 36554857 PMC9778663

[ref-19] Marques MM Ferreira Santos J Rodrigues R : Mobile augmented reality games towards smart learning city environments: learning about sustainability. *Computers.* 2025;14(7):267. 10.3390/computers14070267

[ref-20] Mezirow J : A critical theory of adult learning and education. *Adult Educ.* 1981;32(1):3–24. 10.1177/074171368103200101

[ref-33] Moore J : Is higher education ready for transformative learning? A question explored in the study of sustainability. *J Transform Educ.* 2005;3(1):76–91. 10.1177/1541344604270862

[ref-21] Nusche D Fuster Rabella M Lauterbach S : Rethinking education in the context of climate change: leverage points for transformative change. (OECD Education Working Papers No. 307). OECD Publishing,2024. 10.1787/f14c8a81-en

[ref-22] Presseau J McCleary N Lorencatto F : Action, Actor, Context, Target, Time (AACTT): a framework for specifying behaviour. *Implement Sci.* 2019;14(1): 102. 10.1186/s13012-019-0951-x 31806037 PMC6896730

[ref-23] Raish V Rimland E : Employer perceptions of critical information literacy skills and digital badges. *College & Research Libraries.* 2016;77(1):87–113. 10.5860/crl.77.1.87

[ref-24] Redman A Wiek A : Competencies for advancing transformations towards sustainability. *Front Educ.* 2021;6: 785163. 10.3389/feduc.2021.785163

[ref-25] Scalabrino C : European sustainability competence framework background document: literature review, analysis of frameworks and proposals. Publications Office of the European Union,2022. 10.2760/378627

[ref-26] Sipos Y Battisti B Grimm K : Achieving transformative sustainability learning: engaging head, hands, and heart. *Int J Sustain High Educ.* 2008;9(1):68–86. 10.1108/14676370810842193

[ref-27] Sousa D Menchaca I Doran P : Towards disruptive and dialogic assessment systems supported by technology. In: J. A. C. Gonçalves, J. L. S. M. Lima, J. P. Coelho, F. J. García Peñalvo, & A. García Holgado (Eds.), *Proceedings of TEEM 2023. TEEM 2023. Lecture Notes in Educational Technology*. Springer, Singapore,2024. 10.1007/978-981-97-1814-6_117

[ref-28] Spaccatini F Riva P Richetin J : From past to present (for a better future): the moderating role of cognitive mindset on spillover effects in environmental behaviors. *Curr Psychol.* 2023;42(18):15858–15873. 10.1007/s12144-022-02917-2

[ref-34] Sterling S : Transformative learning and sustainability: sketching the conceptual ground (2011). In: *Learning and Sustainability in Dangerous Times: The Stephen Sterling Reader*. Agenda Publishing,2024;149–162. 10.1017/9781788216920.011

[ref-29] Sulitest: Sulitest TASK content alignment with the EU greencomp framework.2023. Reference Source

[ref-30] UNESCO: Educational content up close: examining the learning dimensions of education for sustainable development and global citizenship education. UNESCO,2019. 10.54675/SRLJ8178

[ref-31] Wals AEJ : Beyond unreasonable doubt: education and learning for socio-ecological sustainability in the Anthropocene. Wageningen University,2015. Reference Source

