# Supplementary material for: Operationalizing the European sustainability competence framework: Development and validation of learning outcomes for GreenComp
Source: Open Res Eur. 2025 Oct 21;5:203. Originally published 2025 Jul 29. [Version 3] doi: 10.12688/openreseurope.20927.3 (PMC12508731; doi:10.12688/openreseurope.20927.3)
Supplement: Peer review history

# Reviewer report: Reviewer response for version 3

**DOI:** 10.21956/openreseurope.23421.r61497

**Published:** 2025-11-08

**Authors:** Tom KUPPENS

**Affiliations:** 1 UHasselt – Hasselt University, Agoralaan, Belgium

The authors operationalised the European sustainability competence framework GreenComp into a validated set of observable, measurable and assessable learning outcomes. The article is one of the outcomes of the Erasmus funded OpenPass4Climate project. The authors adopted a four-phased approach, consisting of (1) a multistakeholder co-design process, (2) followed by a modified Delphi for expert validation, (3) analysis of the full coverage of the GreenComp framework, and (4) a final validation round with experts for the full set of adjusted learning outcomes. This approach resulted in a set of learning outcomes and corresponding assessment criteria that can mainly be used educational stakeholders in upper secondary, vocational and higher education in Western and Southern Europe. Moreover, the authors critically discuss the usability of the assessment tools and its potential risks within the context of open badge initiatives.

 The article contains several strengths, including its roadmap to contextualise and develop the assessment tool even further, for instance, w.r.t. different educational cultures, education levels, disciplines or regional contexts. Moreover, the assessment tool goes beyond the tradition of evaluating the cognitive learning outcomes only, by also proposing attitudinal and behavioural learning outcomes and corresponding assessment criteria. Moreover, the authors address the very important tension between the granularity and holism of assessing sustainability competences. Another strength consists of the collaborative multistakeholder process which increases the potential uptake by and impact among educational practitioners.

 I would like to suggest the following improvements:

- The authors use the concept of ‘education for sustainable development’ which is contested because of the negative connotations of the word ‘development’. Since their approach is student-centred, why not use the concept of ‘learning for sustainability’ proposed by the European Commission?
- The translation of the first sentence of the ‘Métodos’ section in Spanish does not fully correspond to the English version. The authors are recommended to check consistency of the translation.
- Some descriptions of author roles seem to be rather project roles (e.g. project admin) and hence seem to be of little relevance in the contributions section.
- The authors refer to several EU competence frameworks for lifelong learning. It might be inspirational to also mention the FinComp framework because of its progression model, which might reflect potential next steps for building on the authors’ work on the GreenComp operationalisation.
- In the introduction, the authors mention that educational institutions fundamentally shifted the conceptualisation of their role, however, it is mainly experts and policy makers who advocate for this shift. The next step is for educational institutions to implement this, but this is far from reality yet. The authors might want to rephrase the first sentence of their introduction.
- When referring to Sulitest, it is unclear what the alignment percentages mean. The authors might want to elaborate on the meaning and determination of these percentages.
- Other projects that have been working on GreenComp assessment and roadmaps, include GreenScent and ECF4CLIM. Moreover, also the IMPACT project (impactforaction.eu) on impact assessment of the action competence for sustainability might be of interest here. The authors also refer to assessment scales such as SCMM, however, they do not refer to the sustainability consciousness questionnaire (SCQ) of Gericke et al. (2019) yet. I would also like to make the authors aware of developments in the assessment of transformative learning by referring to “A review of transformative learning assessment tools” published by Ngan Ha Nguyen, Thuy Nga Nguyen and Thi Thuy Tang in the Proceedings of the 4 ^th^ Hanoi forum on pedagogical and educational sciences. It might be relevant to refer to these projects, scales and assessment instruments too in the section on “Current state of GreenComp assessment”.
- The authors often refer to several tensions. One tension that has not been mentioned yet, is the tension between the normative character of sustainability education and the danger of a too instrumental approach towards sustainability education. It would be highly appreciated if the authors can reflect on how their assessment instrument address this tension as well.
- The authors use Bloom's taxonomy to operationalize the diverse KSAs of GreenComp. However, Bloom's taxonomy is mostly known for his taxonomy for cognitive learning activities. One advantage is that it probably helped to make the knowledge (K) descriptions more observable/measurable. Although this can be helpful for developing observable learning outcomes for the knowledge (K) and skills (S) of GreenComp, it might be more problematic for the attitudes (A). Can you clarify how a the use of Bloom's taxonomy might have influenced the learning outcomes? Could you recommend taxonomies that might be helpful for the more attitudinal dimensions of GreenComp?
- The authors adopted a modified Delphi approach. It is recommended to explain what the ‘modified’ dimension of the modified Delphi consisted of. How did information from the literature review or the introduction determine the questions asked in the Delphi rounds?
- For the readers and future users of your assessment tool, it would be helpful to add the matrix that maps each of the KSA statements with the final learning outcomes as supplementary material to the article.
- When the authors explain the revisions of the learning outcomes after the first validation round, they provide examples of improved action verbs. However, for some examples the ‘improvement’ is not really clear. For instance, the verb ‘demonstrate’ has been improved to ‘adapt’, whereas these two verbs actually have different meanings. Maybe it is better to provide less examples and provide just one example with its full learning objectives statement to make the improvement clearer.
- Although most learning outcomes in table 1 have a clear connection with sustainability, for some this connection is still missing. Is it possible to reformulate the ones that lack the link to sustainability a little bit in that direction. See for instance the learning outcomes (LO) corresponding to competence 2.2 (LO2), 3.1 (LO2) and 4.3 (LO1). Moreover, it might be relevant to also include the social dimension of sustainability in LO1 of competence 4.2.
- In the discussion, the authors refer to Mezirow’s publication from 1981 to argue that the core aim of EfS must be to transform learners’ perspectives. However, to the best of my knowledge, Mezirow himself did not address sustainability education in that paper. The authors might want to reformulate that statement. It would also be of added value if the authors could more deeply argue why and how their learning outcome assessment tool enables to detect potential change of problematic frames of reference.
- The worked example for a single learning outcome is of high added value for a potential user. The worked example for the integrated assessment, however, lacks the same amount of detail. To make that worked example more useful for potential users, it is recommended to elaborate on the integrated example more deeply.
- When discussing the tensions in the first paragraph of the ‘Challenges and limitations’ section, I would be interested to also see some reflection on the room for open-endedness in transformative sustainability education and how this is reflected in the proposed assessment framework (see also my earlier recommendation to discuss the tension between the normative character of EfS and the risk of a too instrumentalistic approach).

If applicable, is the statistical analysis and its interpretation appropriate?

Yes

Is the study design appropriate and is the work technically sound?

Yes

Is the work clearly and accurately presented and does it engage with the current literature?

Partly

Are the conclusions drawn adequately supported by the results?

Yes

Are sufficient details of methods and analysis provided to allow replication by others?

Yes

Are all the source data and materials underlying the results available?

Partly

Reviewer Expertise:

Learning for sustainability, teacher education

I confirm that I have read this submission and believe that I have an appropriate level of expertise to confirm that it is of an acceptable scientific standard.

# Reviewer report: Reviewer response for version 3

**DOI:** 10.21956/openreseurope.23421.r62568

**Published:** 2025-11-03

**Authors:** Ioan Prigoreanu

**Affiliations:** 1 University of Life Sciences (IULS), Iaşi, Romania

The article is well structured, scientifically rigorous, fully documented, and meets the requirements of an international academic Indexed. It can be considered suitable for Indexed in its current form.

If applicable, is the statistical analysis and its interpretation appropriate?

Partly

Is the study design appropriate and is the work technically sound?

Partly

Is the work clearly and accurately presented and does it engage with the current literature?

Yes

Are the conclusions drawn adequately supported by the results?

Yes

Are sufficient details of methods and analysis provided to allow replication by others?

Partly

Are all the source data and materials underlying the results available?

Yes

Reviewer Expertise:

management, audit, accounting, sustainability,

I confirm that I have read this submission and believe that I have an appropriate level of expertise to confirm that it is of an acceptable scientific standard.

# Reviewer report: Reviewer response for version 3

**DOI:** 10.21956/openreseurope.23421.r62569

**Published:** 2025-10-28

**Authors:** Thais Dibbern

**Affiliations:** 1 State University of Campinas, Campinas, Brazil

My assessment of the revised manuscript is complete. The authors have fully responded to my feedback, resulting in a more robust paper.

If applicable, is the statistical analysis and its interpretation appropriate?

Not applicable

Is the study design appropriate and is the work technically sound?

Yes

Is the work clearly and accurately presented and does it engage with the current literature?

Yes

Are the conclusions drawn adequately supported by the results?

Yes

Are sufficient details of methods and analysis provided to allow replication by others?

Yes

Are all the source data and materials underlying the results available?

Yes

Reviewer Expertise:

Public policy, sustainability, higher education studies

I confirm that I have read this submission and believe that I have an appropriate level of expertise to confirm that it is of an acceptable scientific standard.

# Reviewer report: Reviewer response for version 2

**DOI:** 10.21956/openreseurope.23097.r61505

**Published:** 2025-10-08

**Authors:** Ioan Prigoreanu

**Affiliations:** 1 University of Life Sciences (IULS), Iaşi, Romania

The manuscript presents a significant effort to operationalize the European sustainability competence framework (GreenComp) by developing 40 measurable learning outcomes. The authors build upon the OpenPass4Climate project, employing a four-phase methodology: co-design workshops with stakeholders, a first expert validation (modified Delphi method), systematic coverage analysis, and a second expert validation. The process involved stakeholders from formal and non-formal education, learners at multiple levels, and an international expert panel.

 The outcomes were carefully aligned with GreenComp’s four competence areas and the Knowledge–Skills–Attitudes (KSA) structure, and they were refined to ensure measurability, observability, and student-centered formulation. Importantly, the authors addressed previously identified gaps by adding outcomes on philosophical, ethical, and systemic dimensions, as well as on economic transformation and resource conflict dynamics.

 The article contributes to Education for Sustainable Development (ESD) by moving GreenComp from an abstract policy tool toward practical implementation. The manuscript is well-written, methodologically transparent (particularly in version 2), and highly relevant to the European education and sustainability policy landscape.

 Points for improvement (required for scientific soundness)

 The manuscript presents learning outcomes that are measurable and observable, yet it does not provide sufficient detail on how they can be practically assessed. Without clear assessment tools, the applicability of the outcomes in real educational contexts remains limited.

 Include at least one illustrative example of assessment methods (e.g., portfolio evaluation, project-based assessment, peer and self-assessment, or rubrics) linked to a specific learning outcome, to demonstrate feasibility in practice.

 Although the authors acknowledge cultural constraints, the proposed outcomes are largely framed from a Western European perspective. Considering GreenComp’s intended broad application across Europe and beyond, stronger attention to cultural adaptability is necessary. Explicitly highlight these limitations and propose strategies to adapt the outcomes to diverse cultural, educational, and socio-political contexts, including Eastern European and global perspectives.

 The manuscript appropriately addresses the inherent tension between “gestalt competencies” and measurable outcomes. However, it does not sufficiently illustrate how holistic sustainability competencies can be preserved in assessment and credentialing systems. Provide concrete examples of integrated or constellation-based assessment approaches that could help avoid the fragmentation of competencies into disconnected elements.

 The “Future Directions” section outlines useful research avenues but remains focused primarily on academic implications. To maximize practical relevance, a clearer roadmap for application in educational practice would be beneficial. Expand this section to include specific guidance for educators and policymakers, such as strategies for teacher training, curriculum integration, and progression pathways across different educational levels.

If applicable, is the statistical analysis and its interpretation appropriate?

Partly

Is the study design appropriate and is the work technically sound?

Partly

Is the work clearly and accurately presented and does it engage with the current literature?

Yes

Are the conclusions drawn adequately supported by the results?

Yes

Are sufficient details of methods and analysis provided to allow replication by others?

Partly

Are all the source data and materials underlying the results available?

Yes

Reviewer Expertise:

management, audit, accounting, sustainability,

I confirm that I have read this submission and believe that I have an appropriate level of expertise to confirm that it is of an acceptable scientific standard, however I have significant reservations, as outlined above.

# Response

**Published:** 2025-10-15

**Authors:** Pablo Martín-Ramos

**Affiliations:** Ingeniería Agrícola y Forestal, Universidad de Valladolid, Palencia, Castile and León, Spain

**R3-Q1 & Q2. Practical assessment detail and illustrative example**

 Q1: " *The manuscript presents learning outcomes that are measurable and observable, yet it does not provide sufficient detail on how they can be practically assessed. Without clear assessment tools, the applicability of the outcomes in real educational contexts remains limited.*"

 Q2: *"Include at least one illustrative example of assessment methods (e.g., portfolio evaluation, project-based assessment, peer and self-assessment, or rubrics) linked to a specific learning outcome, to demonstrate feasibility in practice.*"

**Response: **We appreciate this important observation. While Annex I provides detailed specifications for each learning outcome—including observable behaviors, measurable criteria, and suggested evaluation methods—we recognize that concrete worked examples significantly enhance practical applicability for educators. To address this concern, we have added a new "Implementation notes" subsection in the Results section that presents two complementary assessment examples: the first one provides a complete assessment pathway for a single learning outcome, while the second example demonstrates integrated assessment of multiple outcomes through a community sustainability intervention project, illustrating how educators can assess several learning outcomes holistically through a single complex task while maintaining the gestalt nature of sustainability competencies and avoiding fragmentation. Additionally, we have added signposting text in the Results section (before Table 1) that explicitly directs readers to these worked examples and to the comprehensive assessment specifications in Annex I, making the connection between the learning outcomes framework and practical implementation tools more transparent.

Changes made to manuscript: A paragraph has been added to the Results > Final learning outcome distribution, and two examples have been included in the new Results/Implementation notes subsection.

**R3-Q3. Cultural adaptability and contextualization**

 " *Although the authors acknowledge cultural constraints, the proposed outcomes are largely framed from a Western European perspective. Considering GreenComp's intended broad application across Europe and beyond, stronger attention to cultural adaptability is necessary. Explicitly highlight these limitations and propose strategies to adapt the outcomes to diverse cultural, educational, and socio-political contexts, including Eastern European and global perspectives.*"

**Response: **This is a crucial point, and we agree that the framework's Western/Southern European origins must be explicitly acknowledged alongside a concrete strategy for cultural adaptation. We have made two significant additions to address this concern. First, we have added a new subsection to the Discussion, "Cultural adaptability and contextualization", which outlines a four-step protocol for adapting the learning outcomes to diverse contexts. This protocol involves (i) local co-design and stakeholder engagement, (ii) translation and localization of concepts (not just words), (iii) integration of locally salient issues and knowledge traditions, and (iv) equity-by-design checks. Second, we have substantially expanded the Discussion > Limitations section to explicitly state the current geographical scope, identify specific Western assumptions embedded in some outcomes (e.g., regarding political agency, democratic participation, human-nature relationships), and note the need for future comparative validations with partners in Eastern/Northern Europe and non-EU contexts.

Changes made to manuscript: (1) The Discussion > Limitations section has been expanded to elaborate on the cultural scope and outline next steps for validation. (2) A new subsection, "Cultural adaptability and contextualization", has been inserted in the Discussion section after "Challenges and limitations". 

**R3-Q4. Holistic competencies and constellation-based assessment**

 " *The manuscript appropriately addresses the inherent tension between 'gestalt competencies' and measurable outcomes. However, it does not sufficiently illustrate how holistic sustainability competencies can be preserved in assessment and credentialing systems. Provide concrete examples of integrated or constellation-based assessment approaches that could help avoid the fragmentation of competencies into disconnected elements.*"

**Response:** This concern is directly addressed through our response to Reviewer 2's Q3, where we provide a detailed worked example of constellation-based assessment demonstrating how multiple learning outcomes can be evaluated holistically through a single complex task using cross-cutting rubric criteria. Additionally, the critical analysis of micro-credentialing risks and safeguards (our response to R2-Q4) explicitly discusses constellation-based credentialing as a strategy to preserve gestalt competence while meeting recognition requirements. Together, these additions provide both concrete examples and conceptual frameworks for avoiding fragmentation.

Changes made to manuscript: (1) Integrated assessment example as detailed in response to R2-Q3; (2) critical micro-credentialing section as detailed in response to R2-Q4. 

**R3-Q5. Practical roadmap for educators and policymakers**

 " *The 'Future Directions' section outlines useful research avenues but remains focused primarily on academic implications. To maximize practical relevance, a clearer roadmap for application in educational practice would be beneficial. Expand this section to include specific guidance for educators and policymakers, such as strategies for teacher training, curriculum integration, and progression pathways across different educational levels.*"

**Response:** This is an excellent recommendation to enhance the practical relevance of our work. We have substantially revised and expanded the "Future Directions" section to provide concrete, actionable guidance across five key implementation domains: (1) educator capacity building through practice-based professional development; (2) curriculum integration strategies emphasizing transdisciplinary infusion; (3) staged progression pathways across educational levels; (4) quality assurance through calibration and moderation; and (5) institutional support requirements including resource allocation and policy alignment. This transforms the section from a primarily research-focused agenda into a practical implementation roadmap for practitioners and policymakers, while maintaining appropriate discussion of needed research.

Changes made to manuscript: The opening section of Discussion > Future Directions has been replaced with a comprehensive five-point practice roadmap.

# Reviewer report: Reviewer response for version 2

**DOI:** 10.21956/openreseurope.23097.r61501

**Published:** 2025-10-08

**Authors:** Thais Dibbern

**Affiliations:** 1 State University of Campinas, Campinas, Brazil

Dear Authors,

 While the methodology is improved in Version 2, a few points could be sharper:

- **Expert Panel Selection:** The criteria are good, but stating the *number* of publications or years of experience would add objectivity. The gender balance (8 women, 4 men) is noted, but a brief mention of geographical distribution (beyond "diverse institutional contexts") would strengthen the claim of European representativeness.
- **Co-design Synthesis:** The process of merging "similar outcomes" and using "internal Delphi surveys" is described but could be more transparent. A brief example of what made outcomes "similar" and what criteria were used in the internal ranking would be useful.

About the discussions:

- The discussion could be strengthened by providing a concrete example of **how two or three different learning outcomes might be assessed together in a single, complex task** (e.g., a community project) to demonstrate how the "gestalt" can be preserved in practice.
- The section on digital badges is somewhat optimistic. It could more forcefully address the significant risk of **trivialization**—where profound competencies are reduced to a checklist for a badge. A deeper critique of the potential conflict between the micro-credentialing logic and the transformative, holistic aims of ESD would be valuable.

Furthermore, the abstract could be even more impactful by stating the *number* of experts (12) and stakeholders (48) involved, highlighting the scale of the validation process. Another recommendation is to **add a visual element** to the results, such as a chart summarizing the coverage analysis findings.

If applicable, is the statistical analysis and its interpretation appropriate?

Not applicable

Is the study design appropriate and is the work technically sound?

Yes

Is the work clearly and accurately presented and does it engage with the current literature?

Yes

Are the conclusions drawn adequately supported by the results?

Yes

Are sufficient details of methods and analysis provided to allow replication by others?

Yes

Are all the source data and materials underlying the results available?

Yes

Reviewer Expertise:

Public policy, sustainability, higher education studies

I confirm that I have read this submission and believe that I have an appropriate level of expertise to confirm that it is of an acceptable scientific standard, however I have significant reservations, as outlined above.

# Response

**Published:** 2025-10-15

**Authors:** Pablo Martín-Ramos

**Affiliations:** Ingeniería Agrícola y Forestal, Universidad de Valladolid, Palencia, Castile and León, Spain

**R2-Q1. Expert panel selection: objectivity and geography**

 " *Expert Panel Selection: The criteria are good, but stating the number of publications or years of experience would add objectivity. The gender balance (8 women, 4 men) is noted, but a brief mention of geographical distribution (beyond 'diverse institutional contexts') would strengthen the claim of European representativeness*."

**Response:** We agree that providing objective thresholds and an explicit geographical distribution of the expert panel enhances the transparency and credibility of our validation process. The Methods section has been revised to include specific eligibility criteria for the experts (≥5 years of relevant practice and/or ≥5 relevant peer-reviewed outputs) and to list the countries where the experts are currently working (Sweden, Greece, Portugal, Spain, Italy, and North Macedonia). Furthermore, we have added a statement to the Discussion's Limitations section to explicitly acknowledge that this distribution skews toward Southern and Western Europe and have committed to broadening this representation in future validation cycles.

Changes made to manuscript: (1) The first paragraph of Methods > Phase 2: First expert validation round has been expanded to include the objective thresholds, gender balance, sectoral mix, and country distribution. (2) The Discussion > Limitations section has been updated to explicitly note the regional skew.

**R2-Q2. Co-design synthesis: transparency**

 " *Co-design Synthesis: The process of merging 'similar outcomes' and using 'internal Delphi surveys' is described but could be more transparent. A brief example of what made outcomes 'similar' and what criteria were used in the internal ranking would be useful*."

**Response: **We agree that transparency is crucial for the methodological rigor of the consolidation phase. To clarify this process, we have amended the Methods section to include a concrete example of how two draft outcomes were merged because they targeted the same set of GreenComp Knowledge-Skills-Attitudes (KSAs) and had overlapping observable evidence. We have also explicitly stated the three criteria used for the internal ranking—(i) precise alignment to KSA granularity, (ii) clarity of measurability/observability, and (iii) non-duplication—and the tie-breaking protocol, making the synthesis logic both auditable and reproducible.

Changes made to manuscript: An example of the merging process and the explicit internal ranking criteria have been added to the end of the last paragraph of Methods > Phase 1: Initial co-design process.

**R2-Q3. Preserving gestalt in integrated assessment**

 " *The discussion could be strengthened by providing a concrete example of how two or three different learning outcomes might be assessed together in a single, complex task (e.g., a community project) to demonstrate how the 'gestalt' can be preserved in practice.*"

**Response: **This is an excellent recommendation that addresses the central challenge of operationalizing competencies without losing their holistic nature. To demonstrate how the "gestalt" of sustainability competence can be preserved in practice, we have added an " Integrated assessment example" in the new Results/Implementation notes section. This example details how a single, complex task—a community sustainability intervention project—can be used to assess three distinct learning outcomes from different GreenComp areas (Systems Thinking, Futures Literacy, and Collective Action) simultaneously, using an integrated portfolio and a single analytic rubric with cross-cutting criteria rather than separate checklists.

Changes made to manuscript: A new example has been added to the Results/Implementation notes section, immediately after the ‘Worked example (single outcome)’, to illustrate an integrated, multi-outcome assessment task.

**R2-Q4. Micro-credentials and the risk of trivialization**

 " *The section on digital badges is somewhat optimistic. It could more forcefully address the significant risk of trivialization—where profound competencies are reduced to a checklist for a badge. A deeper critique of the potential conflict between the micro-credentialing logic and the transformative, holistic aims of ESD would be valuable*."

**Response:** We fully agree with this critical observation and have revised the Discussion to move beyond an optimistic framing of digital badges. A new subsection has been added to critically examine the structural tensions between the logic of micro-credentialing and the transformative, holistic aims of ESD. This section explicitly addresses the risks of "badge inflation," where credentials are issued without rigor, and "checklist drift," where assessment degrades from authentic competence demonstration to mere participation. To counter this, we propose a series of concrete governance and quality assurance safeguards, including rigorous evidence standards tied to authentic performance, the use of constellation-based credentialing for integrated competencies, transparent issuer accountability through detailed metadata, systematic moderation and calibration protocols, and the embedding of badge acquisition within transformative pedagogical approaches rather than as a replacement for them.

Changes made to manuscript: A new subsection, "Critical perspective: Micro-credentialing risks and necessary safeguards", has been inserted in the Discussion section.

**R2-Q5. Abstract enhancement with participant numbers**

 " *Furthermore, the abstract could be even more impactful by stating the number of experts (12) and stakeholders (48) involved, highlighting the scale of the validation process.*"

**Response: **We agree that including the specific number of participants in the abstract immediately conveys the scale of our validation work. The abstract has been revised to state that the process involved co-design workshops with 48 stakeholders and validation by a 12-member expert panel.

Changes made to manuscript: The second sentence of the Abstract has been revised to include the numbers of stakeholders and experts. Translations into French, German, Italian, and Spanish have been updated accordingly.

**R2-Q6. Add a visual on coverage analysis**

 " *Another recommendation is to add a visual element to the results, such as a chart summarizing the coverage analysis findings*."

**Response: **We agree that a visual element would enhance the accessibility and impact of the coverage analysis findings. In response to this suggestion, we have added a new figure (Figure 1) to the Results section. This figure shows the analytical progression from our coverage assessment to the concrete enhancement actions taken, providing readers with an at-a-glance understanding of the evidence-based process that strengthens the methodological rigor of our work.

Changes made to manuscript: “Figure 1. Coverage analysis: from gaps to actions" has been inserted in the Results section after the coverage analysis findings paragraph; the transition text in the "Enhancement strategies" subsection has been modified to explicitly reference the figure; streamlined redundant numerical details in the text to avoid duplication with the figure.

# Reviewer report: Reviewer response for version 2

**DOI:** 10.21956/openreseurope.23097.r59619

**Published:** 2025-09-15

**Authors:** Voicu-Dan Dragomir

**Affiliations:** 1 The Bucharest University of Economic Studies, Bucharest, Romania

The authors have answered my recommendations regarding the methodological details necessary to bring credibility to the research.

If applicable, is the statistical analysis and its interpretation appropriate?

Not applicable

Is the study design appropriate and is the work technically sound?

Partly

Is the work clearly and accurately presented and does it engage with the current literature?

Yes

Are the conclusions drawn adequately supported by the results?

Partly

Are sufficient details of methods and analysis provided to allow replication by others?

No

Are all the source data and materials underlying the results available?

Partly

Reviewer Expertise:

Sustainability disclosure and environmental assessment.

I confirm that I have read this submission and believe that I have an appropriate level of expertise to confirm that it is of an acceptable scientific standard.

# Reviewer report: Reviewer response for version 1

**DOI:** 10.21956/openreseurope.22641.r57690

**Published:** 2025-09-02

**Authors:** Voicu-Dan Dragomir

**Affiliations:** 1 The Bucharest University of Economic Studies, Bucharest, Romania

The method for creating the competence framework is described, but there are a lot of details missing from this paper. For example, how were the workshops organized, who were the experts (as demographics) and how were they selected, how was the validation conducted etc. Also, the methods need to be explained as well: coverage analysis (for example), what did that entail? In the end, just the finished product is presented, but the method does not show the process in sufficient detail to make it credible or reproducible.

If applicable, is the statistical analysis and its interpretation appropriate?

Not applicable

Is the study design appropriate and is the work technically sound?

Partly

Is the work clearly and accurately presented and does it engage with the current literature?

Yes

Are the conclusions drawn adequately supported by the results?

Partly

Are sufficient details of methods and analysis provided to allow replication by others?

No

Are all the source data and materials underlying the results available?

Partly

Reviewer Expertise:

Sustainability disclosure and environmental assessment.

I confirm that I have read this submission and believe that I have an appropriate level of expertise to confirm that it is of an acceptable scientific standard, however I have significant reservations, as outlined above.

# Response

**Published:** 2025-09-02

**Authors:** Pablo Martín-Ramos

**Affiliations:** Ingeniería Agrícola y Forestal, Universidad de Valladolid, Palencia, Castile and León, Spain

We thank Dr. Dragomir for the careful review and constructive feedback. We acknowledge that greater methodological transparency is essential for credibility and reproducibility. We have substantially expanded the Methods section to provide comprehensive details on workshop organization, participant and expert selection, validation procedures, and coverage analysis methodology. Below we respond point-by-point to the Reviewer’s observations and list the manuscript insertions that we have made.  

**Comment 1: Workshop organization** Reviewer’s comment: “ *how were the workshops organized…*” Response: Thank you for highlighting the need for more detail on the co-design workshops. This initial phase was a complex, multi-stakeholder process that warrants a more thorough description. We have expanded the Methods section to provide comprehensive details on the workshop structure and process. The participant composition and synthesis process are now fully detailed. Changes made to manuscript: [ *Section: Methodology, Subsection: Phase 1: Initial co-design process*] “The co-design phase represented a deliberate departure from expert-driven approaches to learning outcome development. Rather than beginning with technical specifications derived from learning theory, we initiated a collaborative process across the four OpenPass4Climate consortium institutions. Each institution assembled carefully balanced stakeholder groups: (1) at least three educators from formal education (university faculty at UniLaSalle, NOVA, and University of Valladolid; vocational trainers at CSCI Novara); (2) at least three practitioners from non-formal settings, recruited from corporate sustainability professionals engaged in the project's ‘Work Package 5 - Impact evaluation, enlargement & policy recommendations’); and (3) at least five learners representing different educational levels, such as vocational and A-level students (CSCI Novara), Bachelor's and Master's students (UniLaSalle, NOVA), and doctoral candidates (University of Valladolid). Learner participants were volunteers selected via a convenience sampling approach from those involved in the prior surveys and focus groups of the project’s ‘Work Package 3 - Nature & assessment of engagement' (Martín-Ramos et al., 2025). By bringing together these diverse perspectives (spanning educational levels, professional contexts, and implementation settings), we ensured the resulting learning outcomes would be meaningful across the full spectrum of contexts where GreenComp might be applied. The co-design process unfolded through a series of structured workshops conducted over three months. Each institution hosted four workshops (one per GreenComp competence area) online via Microsoft Teams and Miro collaborative platform, conducted in local languages without time constraints to facilitate nuanced discussion. Participants worked in interdisciplinary groups to translate abstract KSA statements into concrete learning outcomes. To structure the KSA translation process, we employed Bloom's revised taxonomy (Anderson & Krathwohl, 2001) as a heuristic device rather than a rigid framework. Participants were encouraged to consider how each competence might manifest at different cognitive levels, from basic comprehension to creative synthesis. However, we found that many sustainability competencies resisted easy categorization within Bloom's framework, particularly those involving affective dimensions or systems thinking. This led to iterative refinement of outcomes that attempted to capture the holistic nature of competencies while maintaining sufficient specificity for assessment purposes. The learning outcomes were formulated according to the Centre Européen pour le Développement de la Formation Professionnelle (CEDEFOP) guidelines for defining, writing and applying learning outcomes (CEDEFOP, 2022), ensuring they were observable (describing a behavior or product that can be observed), measurable (assessable using specific criteria), and student-centered (focusing on what the learner will be able to do). Each outcome was designed to integrate relevant knowledge, skills, and attitudes from the GreenComp framework, translating the abstract KSAs into concrete statements of what learners should demonstrate. Following the institutional workshops, a cross-consortium synthesis process was undertaken by project staff (without participation from learners or non-formal trainers) to consolidate the proposals. Translation of outputs from the local languages into English was validated by bilingual consortium members to preserve conceptual accuracy. Similar outcomes were merged, and internal Delphi surveys were used to rank and select the top three learning outcomes for each of the 12 competencies. The initial co-design phase produced 36 learning outcomes, three for each of the 12 GreenComp competencies.”  

**Comment 2: Expert demographics and selection** Reviewer’s comment: “ *who were the experts (as demographics) and how were they selected…*” Response: We agree that providing more information on the expert panel enhances the credibility of the validation phase. We have added details about expert panel composition and selection criteria. Changes made to manuscript: [ *Section: Methodology, Subsection: Phase 2: First expert validation round*] “The expert validation phase employed a modified Delphi approach designed to balance quality assurance with practical constraints, such as the project timeline and expert availability. Our expert panel comprised 12 specialists (8 women, 4 men) selected based on demonstrated expertise in sustainability education and familiarity with European competence frameworks. Selection criteria included authorship of GreenComp-related publications or reports, active engagement in the GreenComp Community, and participation in EU sustainability education projects such as TWIN-IN, ASSESS, EduC3, GrowLearnOut, GreenScent, GreenAtYou, and Entrepreneurship4All. The resulting panel represented diverse institutional contexts: five from higher education institutions, five from NGOs focused on sustainability education, one independent researcher, and the GreenComp Community manager. This institutional diversity proved crucial during the validation process, as experts brought culturally and contextually specific insights that challenged the universalist assumptions embedded in our initial formulations. […]”  

**Comment 3: Validation process details** Reviewer’s comment: “ *how was the validation conducted*…” Response: We appreciate the reviewer’s suggestion for greater specificity, as this detail strengthens both the credibility and reproducibility of the process. We have clarified the validation methodology, including tools, timelines, and analysis procedures. Changes made to manuscript: [ *Section: Methodology, Subsection: Phase 2: First expert validation round*] “[…] The validation was conducted asynchronously using anonymous Microsoft Forms following an initial briefing via Teams. One form was created per competence area, with experts evaluating each learning outcome through a binary choice: 'valid as is' or 'not valid/requires improvement'. Selection of the latter option triggered a text field for detailed feedback and improvement suggestions. Forms remained open for four weeks to allow sufficient time for thoughtful evaluation. Each learning outcome was assessed against four criteria:

- Alignment: Clear relationship to the target GreenComp competence
- Measurability: Use of clear, assessable verbs and specific performance indicators
- Student-centricity: Focus on learner capabilities rather than teaching activities
- Observability: Potential for demonstration through observable behaviors or products

Analysis followed a conservative protocol: any learning outcome marked as 'not valid/requires improvement' by at least one expert was flagged for revision, regardless of overall approval rates. This approach ensured that all expert concerns were addressed. Qualitative feedback was analyzed thematically to identify recurring concerns and suggestions for improvement, with particular attention to cases where experts disagreed on validity judgments.” [ *Section: Methodology, Subsection: Phase 4: Second expert validation round, second paragraph*] “[…] The second validation round followed a more focused protocol. Unlike the anonymous first round, experts received personalized documentation packages via email containing:

- Detailed rationales for each proposed modification or addition
- Explicit mapping of how changes addressed prior comments or coverage gaps
- Comparison between the original and revised formulations

Experts provided direct, non-anonymous feedback within two weeks, enabling iterative clarification where needed. This approach facilitated deeper engagement with contentious additions, particularly learning outcomes challenging anthropocentric perspectives or addressing resource conflict dynamics. Experts were asked to evaluate not only the technical quality of these outcomes but also their appropriateness within the GreenComp framework and potential implementation challenges.”  

**Comment 4: Coverage analysis methodology** Reviewer’s comment: “ *coverage analysis (for example), what did that entail?*” Response: Thank you for this question. The coverage analysis was a systematic verification process. Its purpose was to ensure our set of learning outcomes comprehensively addressed every single KSA statement outlined in the official GreenComp framework document. This step was critical to prevent any inadvertent omissions and to guarantee that our operationalization was faithful to the original framework's complete scope. We have added a clarifying sentence to the first paragraph of the "Phase 3: Coverage analysis" section to make its purpose explicit. Changes made to manuscript: [ *Section: Methodology, Subsection: Phase 3: Coverage analysis, first paragraph*] “Following the first validation round, we conducted a systematic coverage analysis to assess how comprehensively the learning outcomes addressed all aspects of the GreenComp framework. The primary goal was to verify that our 36 proposed learning outcomes collectively operationalized every individual KSA statement in the official GreenComp publication, ensuring no competence element remained unaddressed. […]”
